# novPTMenzy: a database for enzymes involved in novel post-translational modifications

**DOI:** 10.1093/database/bav039

**Published:** 2015-04-29

**Authors:** Shradha Khater, Debasisa Mohanty

**Affiliations:** Bioinformatics Centre, National Institute of Immunology, Aruna Asaf Ali Marg, New Delhi 110067, India

## Abstract

With the recent discoveries of novel post-translational modifications (PTMs) which play important roles in signaling and biosynthetic pathways, identification of such PTM catalyzing enzymes by genome mining has been an area of major interest. Unlike well-known PTMs like phosphorylation, glycosylation, SUMOylation, no bioinformatics resources are available for enzymes associated with novel and unusual PTMs. Therefore, we have developed the novPTMenzy database which catalogs information on the sequence, structure, active site and genomic neighborhood of experimentally characterized enzymes involved in five novel PTMs, namely AMPylation, Eliminylation, Sulfation, Hydroxylation and Deamidation. Based on a comprehensive analysis of the sequence and structural features of these known PTM catalyzing enzymes, we have created Hidden Markov Model profiles for the identification of similar PTM catalyzing enzymatic domains in genomic sequences. We have also created predictive rules for grouping them into functional subfamilies and deciphering their mechanistic details by structure-based analysis of their active site pockets. These analytical modules have been made available as user friendly search interfaces of novPTMenzy database. It also has a specialized analysis interface for some PTMs like AMPylation and Eliminylation. The novPTMenzy database is a unique resource that can aid in discovery of unusual PTM catalyzing enzymes in newly sequenced genomes.

**Database URL**: http://www.nii.ac.in/novptmenzy.html

## Introduction

**Figure 1.**
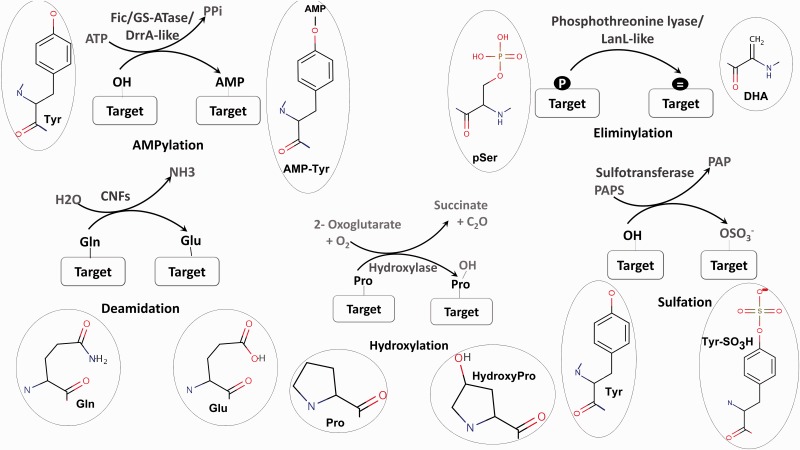
Schematic representation of five unusual PTMs of proteins. For each PTM chemical structures of the amino acid and modified amino acid have been depicted.

Post-translational modifications (PTMs) of proteins are a crucial strategy used by both prokaryotes and eukaryotes to modulate and regulate cellular processes. Modification of proteins can range from the addition of a small chemical moiety such as phosphate to the addition of peptides like ubiquitin and SUMO or the covalent cleavage of peptide backbone (1). The modifications play a central role in intracellular signaling; signaling pathways associated with host-pathogen interactions (2) as well as the biosynthesis of many bioactive natural products like lantibiotics (3) and so enables proteins to acquire new functions. Therefore, the identification of enzymes involved in novel PTMs by genome mining has become an area of major interest. The exponential increase in genome sequences and the experimental characterization of a large number of amino acid modifications in proteins has created a bottleneck in connecting known PTMs to the genes catalyzing them (4). Therefore, it is necessary to decipher the various biochemical pathways associated with PTM catalyzing enzymes by *in silico* genome analysis. PTMs like phosphorylation, glycosylation, SUMOylation have been characterized extensively and a number of bioinformatics tools are available for analysis of the enzymes involved in their catalysis. O-GLYCBASE (5) and Phosphso.ELM (6) are some examples of databases associated with specific classes of PTMs. In contrast to these well-known PTMs, no user friendly tools are available for the identification and analysis of enzymes associated with newly discovered novel PTMs (Figure 1) like AMPylation (7) and Eliminylation (8) and unusual PTMs like Sulfation (9), Hydroxylation (10) and Deamidation (11). Even though these PTMs occur less frequently, they play a crucial role in structural and functional diversification of the proteome and their role in expanding the metabolic and signaling capacities of an organism cannot be underestimated (12).

### AMPylation

AMPylation or adenylylation is the covalent transfer of the AMP moiety from ATP to the hydroxyl group of a tyrosyl or threonyl residue in a protein (Figure 1). Three separate families of enzymes, Filamentation induced by cAMP (Fic) (13), Glutamine Synthetase Adenyl Transferases (GSATase) (14) and Defects in Rab1 recruitment protein A (DrrA) (15) are known to catalyze the AMPylation reaction. GSATase inhibits the activity of Glutamine Synthetase (GS) in the nitrogen metabolism pathway by AMPylation of tyrosine residues (14). AMPylation by Fic and DrrA domains has been shown to be involved in the modification of host proteins by virulent pathogens (13, 15, 16). This regulation of both metabolic and host pathogen interaction pathways by AMPylation makes it a topic of special interest. Involvement of Fic domains in neurotransmission in glial cells (17) and the presence of this domain in multicellular eukaryotes (18) suggest that AMPylation could also have implications for regulation of other biological processes. Computational studies have shown that death on curing (Doc) proteins share sequence similarity with Fic proteins and hence the Fic family is quite often annotated as the Fic/Doc family (18, 19). Apart from AMPylation, the members of Fic/Doc family have been shown to catalyze phosphorylation and phosphocholine transfer (20). Therefore, it is necessary to identify sequence and structural features which distinguish AMPylators from other non-AMPylating family members. Results from biochemical studies and information from available three-dimensional (3D) structures have helped in elucidation of the active site residues and other mechanistic details of AMPylation domains (21–23). Recent studies have also revealed that how inhibitory helices in Fic domains or in their genomic neighbors inhibit AMPylation (21, 24).

### Eliminylation

Eliminylation is another novel PTM associated with both host pathogen interaction (25–27) and biosynthetic pathways (28, 29). Eliminylation involves β elimination of the phosphate group of phosphoserine (pS) or phosphothreonine (pT) converting these amino acids into dehydroalanine (DHA) or dehydrobutyrine (DHB) (Figure 1). Removal of phosphate by β elimination is catalyzed by novel phosphothreonine lyase enzymes present in pathogenic bacteria like *Shigella*, *Pseudomonas syringae* and *Salmonella* (25–27). These bacterial phosphothreonine lyases catalyze the irreversible PTM of host Mitogen-activated protein kinases (MAPKs) by eliminylation of functionally crucial phosphothreonines, converting them to DHB. Since these MAPKs cannot be phosphorylated again, this pathogen-mediated PTM results in the inhibition of the host MAPK signaling pathways. Phosphothreonine lyase domains are also present in LanL like type III and type IV lanthipeptide synthetases along with kinase domains (28). During the biosynthesis of lanthipeptides, these LanL like phosphothreonine lyase domains act on phosphorylated serine/threonine-rich peptides to produce dehydro amino acids like DHA and DHB. DHA/DHB are subsequently cyclized with cysteine residues by lanthionine cyclase enzymes to generate lanthionine groups (28, 29) and so these enzymes are also involved in biosynthesis of natural product lantibiotics. Even though experimental studies so far have only identified phosphothreonine lyases in prokaryotic organisms, based on bioinformatics studies using profile-based searching, fold prediction and genomic neighborhood analysis, we have recently predicted a phosphothreonine lyase function for BLES03 proteins in humans (30).

### Hydroxylation

Collagen chains, the major component of animal tissues, are heavily hydroxylated, majorly at proline and to a lesser extent lysine residue (10, 31). Disruption of PTMs in collagen has been shown to be linked to certain forms of osteogenesis imperfecta and might be linked to ocular and renal pathologies (10). Hydroxylation of proline and asparagine residues is also known to regulate the hypoxia inducible transcription factor (HIF) (32, 33). HIF induces transcription of numerous genes to respond to reduced oxygen levels. The same enzyme, Factor inhibiting HIF (FIH), is also known to regulate ankyrin repeat containing proteins through asparagine and aspartic acid hydroxylation and hence modulate its interactions with other proteins (34). FIH has also been shown to hydroxylate histidine residues in ankyrin repeat domain of tankyrase-2 and could probably be involved in modulation of hypoxic response (35). Thus, aspartic acid or asparagine hydroxylating enzymes can also hydroxylate histidine when it is presented in an appropriate substrate context. Histidine hydroxylation is also catalyzed by a new class of 2-oxoglutarate (2OG)—dependent oxygenase—ROX (36, 37). Human MYC-induced nuclear antigen (MINA53) and Nucleolar protein 66 (NO66) have been shown to catalyze hydroxylation on histidine residue within ribosomal proteins Rpl27a and Rpl8, respectively (36, 37). *Escherichia coli* homolog YcfD has been shown to catalyze hydroxylation on arginine residue of ribosomal protein Rpl16 (36–38). Therefore, enzymes catalyzing hydroxylation of protein residues constitute another important class of PTM catalyzing enzymes.

### Deamidation

In *E. coli* Cytotoxic Necrotizing Factors CNF1, CNF2, CNF3 and CNFγ cause the deamidation of a glutamine residue to a glutamate, thereby constitutively activating host the RhoGTPases (Figure 1) (11). Deamidation by CNF1 from Uropathogenic *E. coli* has been implicated in infections of the urinary tract while CNF2 has been demonstrated to cause diarrhea in calves and lambs (11). Recently, it was shown that CNFγ induces apoptosis in a prostate cancer cell line, making it a potential candidate for the treatment of prostate cancer (39). Tissue transglutaminase-mediated deamidation of glutamine from gluten peptides occurs in celiac disease (40). Transglutamination-mediated transamidation of glutamine residues is also known to be coupled with deamidation (41). Therefore, involvement of deamidation in various infections, diseases and treatment of cancer are also topics of great interest to the scientific community.

### Sulfation

The covalent transfer of sulfate group to tyrosine residues occurs on several proteins like plasma membrane proteins, coagulation factors, adhesion molecules, secretory proteins, immune components and the neuropeptide cholecystokinin (Figure 1) (9). Sulfation helps in modulating the interactions of these proteins and is necessary for their biological activity (42, 43). Therefore, the development of bioinformatics tools for identification of novel sulfotransferases and their substrates is a topic of considerable interest.

The enzymes catalyzing the above-mentioned unusual PTMs and their substrates have been biochemically characterized in recent years. 3D structures for many of these enzymes have also been elucidated by crystallographic studies. This information about sequence, structure, physiological substrates, i.e. particular proteins that these enzymes are known to modify, and the substrate specificity of these PTM catalyzing enzymes is extremely valuable, not only for understanding mechanistic details of these enzymes, but also for identification of such novel enzymes in newly sequenced genomes. Since no databases are available for enzymes which catalyze AMPylation, Eliminylation and Sulfation, protein function annotation resources like Pfam cannot efficiently identify such novel enzymes in newly sequenced genomes. Therefore, we have developed the novPTMenzy database for cataloging sequence, structure and substrate specificity of these unusual PTM catalyzing enzymes. Based on a comprehensive analysis of the sequence and structural features of these enzymes, we have also developed computational tools for identification and classification of these novel enzymes in various genomes. These tools have been made available as a search interface of the novPTMenzy database. The novPTMenzy database has been successfully used to identify several unusual PTM catalyzing domains in proteins with previously unknown function present in newly sequenced genomes.

## Database development

### Data integration and organization

**Figure 2.**
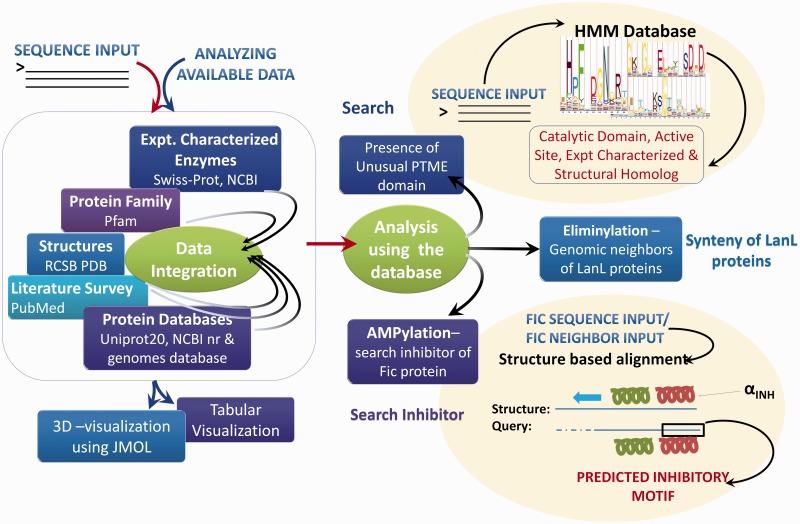
Schema of novPTMenzy workflow. The right panel depicts various possible sequence and structure-based analysis.

**Figure 3.**
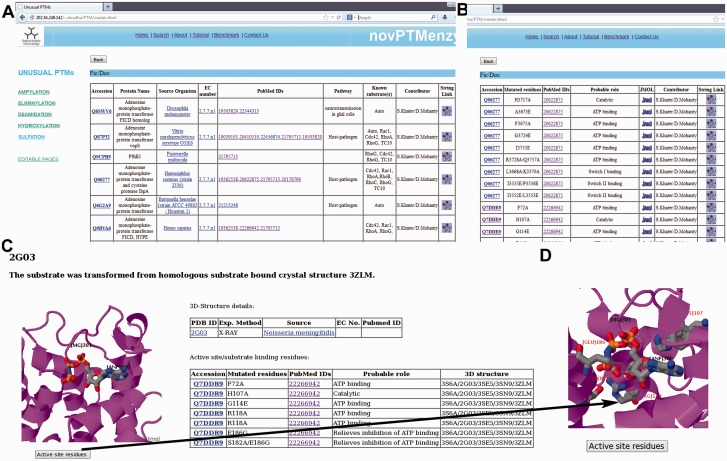
Screenshots depicting typical analysis using novPTMenzy database. (**A**) Table containing experimentally characterized protein. (**B**) Information about active site residue and their role in catalysis based on published experimental studies. (**C**) Graphical visualization of structures along with substrate. (**D**) Pop-up displaying active site residues in stick representation upon clicking the ‘active site residues’ button.

Based on extensive manual curation of published literature, information on sequences, 3D structures, experimentally verified active site residues, native pathways and known substrates for enzymes associated with these five PTMs have been cataloged in the novPTMenzy database (Figure 2). The information on each of these five unusual PTM catalyzing enzymes has been organized in novPTMenzy database into three major sections: (i) Experimentally characterized enzymes (Figure 3A), (ii) Available structures from X-ray crystallographic or NMR studies and (iii) Active site or substrate binding pocket residues (Figure 3B). For each PTM catalyzing enzyme, as well as the amino acid sequence information, other curated information such as the source organism, enzyme commission number, known pathways where these enzymes have been shown to be involved, related literature links and targets that are post-translationally modified by these enzymes are also stored in novPTMenzy database. Available 3D structures of PTM modifying enzymes were downloaded from the Protein Data Bank (PDB) database (44). 3D structures in complex with ligand or ligand transformed from homologous 3D structures were stored in novPTMenzy database. The structural information section of the novPTMenzy database stores structure-related information such as link to individual PDB page, their CATH (45) and SCOP (46) IDs, source organism, PubMed IDs of related literature. The active site or substrate binding pocket residues identified by structural studies as well as mutational analysis have also been compiled in the novPTMenzy database based on a literature survey. Extensive cross-references are provided to various databases such as UniProtKB (47), NCBI taxonomy (48), KEGG (49), PubMed, STRING (50) and PDB (51). The current version of the novPTMenzy database contains information about 73 experimentally characterized unusual PTM catalyzing enzymes from 36 different organisms and 95 crystal or NMR structures available in PDB for enzymes catalyzing unusual PTMs.

### Retrieval and visualization of stored data

Users can browse the database from the menu panel provided on the left side of each page. The menu panel provides links to all the five different PTMs and for each PTM the user can view a detailed report or carry out a number of different analyses using various analysis tools. novPTMenzy provides user-friendly graphical interfaces for the visualization of 3D structures as well as the depiction and analysis of their active site residues using the Jmol applet (Figure 3C). The interface also facilitates visualization of active site residues along with the ligand (Figure 3D). Figure 3D shows the analysis of the 3D structure of the AMPylating enzyme NmFic (PDB ID: 2G03) to highlight the utility of this interface. As can be seen, visualization of active site residues along with transformed ligand not only helps in understanding their role in catalysis, but also highlights how a negatively charged glutamate residue can potentially obstruct the ATP binding site (Figure 3D). This correlates well with experimental evidence showing the glutamate containing helix to be inhibitory and the mutation of the inhibitory glutamate facilitates AMP binding and hence the AMPylation activity by this enzyme (21, 24).

### Editable database

As proteomic and metabolomic data increase exponentially there is a need for quickly incorporating the growing information in our database. In order to accomplish this we have made provision for including data by a crowd-sourcing approach through editable pages. An interested user can incorporate any new information in the respective database page. As community-based data incorporation might impact the data quality, added data are maintained as a separate page and are added to the main database only after careful review by administrators of the novPTMenzy database. By these means, we hope to use crowd-sourcing to keep the novPTMenzy database updated with latest information.

## Development of query interfaces

### Sequence analysis tools

Based on a variety of analyses of sequence and structural information, HMM profiles of the domains catalyzing the five PTMs have been built and stored to be used for various types of predictions in the novPTMenzy database. Since certain PTMs like AMPylation and eliminylation are catalyzed by more than one protein family, 11 different Hidden Markov Models (HMMs) have been derived for enzymes catalyzing these five different PTMs. One of the major advantages of novPTMenzy over other domain identification tools is the presence of HMM profiles for unusual PTM catalyzing enzymes that are not represented in generic databases like Pfam (52). In fact the sequence analysis interface of novPTMenzy cannot only identify different unusual PTM catalyzing domains in a protein sequence, but can also group them into different functional subfamilies using these 11 HMM profiles. For example, a putative AMPylating domain can be classified as Fic, Doc or a phosphocholine transferase.

**Figure 4.**
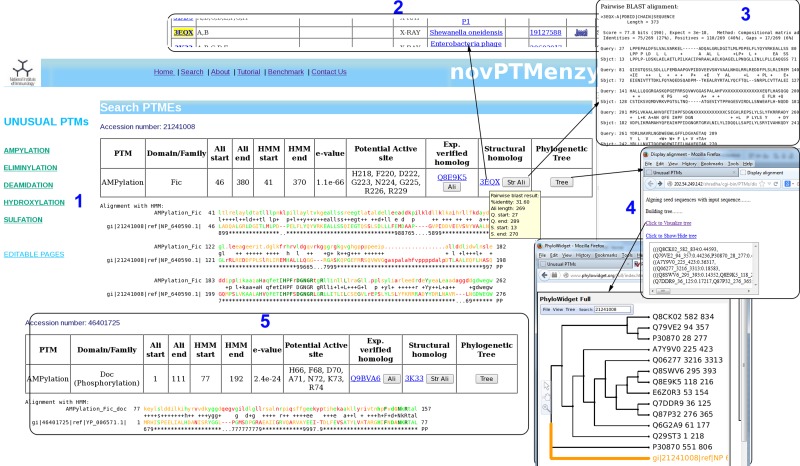
Screenshots depicting typical analysis using search interface and comparative sequence analysis tools of novPTMenzy database. Panel 1: The Search interface used sequence to HMM profile alignment to identify AMPylation domain in query sequence and classified it as Fic type from among Fic, Doc, AvrB and AnkX subfamilies. It also depicts putative active site residues identified in the Fic type AMPylation domain, provides links to experimentally characterized homologs and also structural homologs. Panel 2: Structural homologs of the Fic domain in the query sequence. Panel 3: Alignment with the closest structural homolog obtained by clicking the button labeled ‘Str Ali’ in the structural homolog cell in Panel 1. Panel 4: Tree button in Panel 1 builds a phylogenetic tree of the PTM catalyzing domain in the query sequence along with seed sequences for the corresponding domain stored in novPTMenzy. It could be either visualized by clicking ‘view tree’ button or downloaded for further analysis. Panel 5: Identification of a Doc domain in a different query sequence using the search interface of novPTMenzy.

Given a query sequence (fasta formatted or bare sequence) or an accession number, the search interface of novPTMenzy matches it to profiles of different PTM catalyzing enzymes using HMMER (53) tool. Details of the hit are provided as a table and a color-coded alignment of the sequence with profile is displayed below it (panel 1 in Figure 4). Alignment colors vary from green to red based on the quality of alignment. It also annotates the putative active site residues in the query sequence by highlighting them in the alignment and also displaying them in tabular form (panel 2 in Figure 4). novPTMenzy also provides interfaces for alignment of the query protein sequence with other homologous sequences which have been experimentally characterized as well as with structural homologs present in PDB (panel 3 in Figure 4). A local version of the NCBI BLAST program is used to search for the closest neighbors in the sequence database of experimentally characterized enzymes and enzymes with 3D structures. The accession numbers of closest neighbors displayed in the table are linked back to our database for active site pocket visualization and more annotation. This interface also allows construction of phylogenetic tree and its visualization using a url-based link to the PhyloWidget (54) program (panel 4 in Figure 4). Seed sequences for each PTM catalyzing domains are stored in the novPTMenzy database and are used for construction of the phylogenetic tree using the ClustalW (55) program. Users have an option to download the phylogenetic tree for future offline analyses.

An interesting aspect of novPTMenzy is that the HMM profile-based method combined with the putative active site prediction is used to distinguish between subfamilies of PTM catalyzing enzymes which are functionally divergent. For instance proteins of Fic/Doc family have diverged to perform different functions. Although the Fic subfamily catalyzes the AMPylation reaction, recent studies indicate that members of Doc subfamily are involved in phosphorylation (56) and Ankyrin repeat-containing protein X (AnkX) like proteins are involved in phosphocholine transfer (20). Due to their sequence similarity these proteins are grouped under the Fic family by generic databases like Pfam. However, the search interface of novPTMenzy uses profiles to distinguish between Fic and Doc proteins and active site residues are used to distinguish the Fic subfamily from the Doc and AnkX subfamilies. The sensitivity and specificity of the Fic profile to distinguish between Fic and Doc proteins were 84.07 and 98%, respectively, and the corresponding values for Doc profile were 90 and 92.47%, respectively (Table 1). Figure 4 displays the output from the novPTMenzy search interface when sequentially similar but functionally divergent Fic and Doc proteins are given as input. The interface recognizes Fic and Doc proteins correctly giving the details of the alignment like the *e*-value, start and end position of Fic/Doc domains and the HMM profile used to identify the corresponding domain. Using the predicted active site residues, the differences in the active sites of Fic and Doc proteins can be inferred. Doc proteins have a conserved lysine residue (K73) in place of glycine (G225) residue of Fic proteins. Also, Fic proteins have an extra active site residue (R229) compared with Doc proteins. In addition, the closest experimentally characterized protein and PDB structure is provided. Hovering the mouse over the accession numbers displays some details of the alignment whereas the alignments to closest sequences can be visualized using the clickable buttons.

**Table 1.** Benchmarking the performance of novPTMenzy for identification of different families of PTM catalyzing domains

Statistical parameter	AMPylation			Eliminylation	
	Fic	Doc	GSATase	PTLs	LanL
Sensitivity (%)	84.1	90.0	100	100	100
Specificity (%)	98.0	92.5	100	100	100
PPV (%)	99.5	72.6	100	100	100
Accuracy (%)	86.6	90.9	100	100	100

PPV, Positive Prediction Value; PTLs, Phosphothreonine lyase.

## Additional analysis tools/features

### Search for inhibitory helices of AMPylation domains

**Figure 5.**
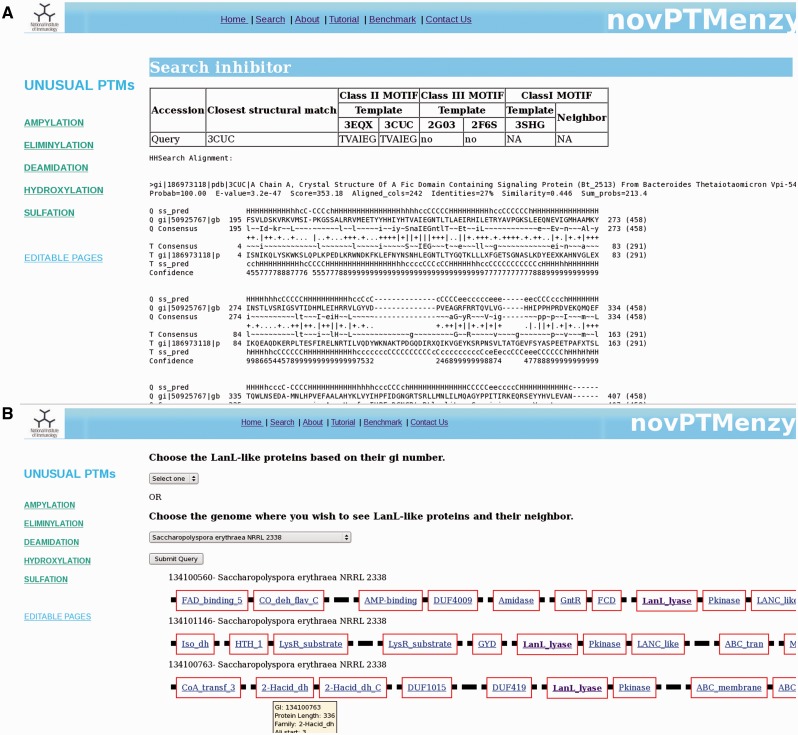
(**A**) Results from ‘Search Inhibitory helix’ interface that predicts inter or intra inhibitory helices of Fic domains. Along with classification of the identified inhibitory helix as class I, II or III, it helps in prediction of inhibitory motif. It also shows the structure-based profile–profile alignment based on which the given inhibitory helix was predicted. (**B**) Screenshot depicting genomic neighborhood of a typical LanL protein containing eliminylation domain. Each gene is represented by a thick black line and the functional domains present in a given gene are depicted by red-colored rectangular boxes with the name of the domain inscribed in the box. novPTMenzy has assigned all functional domains using Pfam database, except for eliminylation domain which has been identified by HMM profiles stored in backend databases of novPTMenzy.

For AMPylation, novPTMenzy provides specific tools for identifying intra or inter inhibitory helices involved in the regulation of AMPylation activity. AMPylation by Fic is known to be regulated by small inhibitory domains present on the same polypeptide chain or on neighboring genes on the genome (21). The inhibitory glutamate in aforementioned helix obstructs the ATP binding site and hence inhibits AMPylation by the Fic domain. Based on the presence of inhibitory helix, Fic proteins are classified as class I, II and III. Class I Fic proteins are regulated by inhibitory helices present in neighboring proteins whereas class II and class III proteins are regulated by inhibitory helices present in N-terminal and C-terminal of the Fic domain, respectively. It was shown that the inhibitory helix contains a conserved motif containing the glutamate. To identify the inhibitory domain either in the Fic protein or in their genomic neighborhood, novPTMenzy uses the structure-based profile–profile comparison tool HHSearch (57). Profile HMMs for all the available Fic/Doc structures were built and stored in the backend database. The additional advantage of HHSearch over other profile-based method is the incorporation of secondary structure information in its profile and use of iterative searches to build them. Also, HHsearch relies on profile–profile comparison rather than sequence–profile comparison. This makes HHSearch more compute intensive but its higher sensitivity allows the detection of short helices with divergent sequence containing the inhibitory glutamate. HHpred profiles were built for class II Fic domain BtFic (PDB ID: 3CUC) and SoFic (PDB ID: 3EQX), class III Fic domains NmFic (2G03) and HpFic (2F6S) and for class I inhibitory protein VbhA (3SHG) present in the genomic neighborhood of VbhT Fic domain. These structure-based sequence profiles were stored in our database along with information about the inhibitory motif. Users have an option of giving either just a Fic protein or Fic protein along with its neighbors (maximum 2). For each input sequence HHPred profiles containing structural information are built. The structural information is based on PSIPRED (58) predicted secondary structure. The profiles corresponding to input Fic proteins are compared with the profiles stored for class II and class III Fic proteins in our database. If the alignment has an *e*-value of <0.001 it is checked for presence of a helix corresponding to inhibitory helix of class II and class III proteins. If the inhibitory glutamate is present in the helix, the query protein is classified as class II or III Fic by novPTMenzy. If the inhibitory glutamate and helix is not located in the Fic protein, profiles of neighbors are aligned to VbhT profile. The Fic protein is labeled as class I based on the presence of inhibitory glutamate in the profile of the neighbor. An option to input the accession numbers of Fic sequences is also available. Accession numbers are mapped to NCBI accession numbers and the sequences are fetched from a locally downloaded nr database. Also, the sequences of its neighbors are retrieved from completely or partially sequenced genomes. novPTMenzy has stored the genomic positions of all proteins from completely sequenced genomes based on information from NCBI’s Mapviewer. A search for inhibitory glutamate is done as described. Figure 5A shows a typical output for inhibitory helix search, when a Fic protein Huntingtin yeast partner E (HYPE) from *Rattus norvegicus* is given as input. The best hit using structure-based profile–profile match is 3CUC and HYPE is classified as a Fic domain containing class II inhibitory helix with a motif TVAIEG (Figure 5A). It may be noted that HYPE from *Homo sapiens* has been experimentally shown to be a Fic domain containing class II inhibitory helix (21). This module of novPTMenzy would help biochemists in designing experiments for detailed study of regulation of Fic domains.

### Synteny of LanL family

LanL, class IV lantibiotic syntheses, along with several other enzymes which co-occur in its genomic neighborhood post-translationally modify ribosomally synthesized small peptides (28, 59). LanL like enzymes have kinase, lyase and cyclase domains fused, which together catalyze the dehydration of serine/threonine involving phosphorylation and eliminylation, and its subsequent cyclization with cysteine. Transformation to mature lanthipeptides also involves PTMs by methyl transferases, acetyl transferases, hydrolases, decarboxylases, etc. Peptidase, transporters and two component regulatory proteins are also involved in its synthesis (28). All these genes along with potential lanthipeptides co-occur in the genomic neighborhood of LanL proteins. Therefore, to understand biosynthesis of lanthipeptides by LanL, it is also important to study its synteny. What makes studying the biosynthesis of these unusually modified peptides fascinating is that they have bactericidal properties and their potential use as new antibiotic.

For a detailed understanding of eliminylation domains in lanthipeptide biosynthesis we have developed the interface ‘Synteny of LanL family’, which helps to analyze the genomic neighborhood of eliminylation domains co-occurring with other enzymes associated with biosynthesis of lanthipeptides. We have collected sequences of the LanL family from the nr database using a novPTMenzy profile. Neighbors of these LanL enzymes were extracted from completely sequenced genomes. To understand their functional significance, the Pfam domain definition for each neighbor was collected and stored in the database. Pfam domain descriptions provide functional insight into the protein of interest. ‘Synteny of LanL family’ uses a backend database of 208 LanL proteins. Of these 208 LanL proteins, genomic neighborhood information was present for only 51 proteins. Five hundred genomic neighbors for these 51 LanL proteins were extracted and stored in the backend database. The interface ‘Synteny of LanL family’ can be accessed from the Eliminylation main page. From this interface the user can choose a single LanL protein or all LanL proteins from an organism using the drop down menu. LanL protein(s) from the chosen organism along with its neighbors is displayed graphically. Also, associated Pfam domains with a link to the Pfam database is provided for each protein*.* Hovering on the link gives the accession number of the neighboring protein and details of its alignment with the Pfam profile. Because the lyase domain of LanL-like proteins is not recognized by Pfam, alignment details of the protein with the novPTMenzy profile is shown by hovering the mouse and the link out is provided to the eliminylation database page of novPTMenzy. Figure 5B shows a typical output containing three LanL proteins when *Saccharopolyspora erythraea* NRRL 2338 is selected for synteny analysis. As can be seen, all three clusters are associated with peptidases and transporters and tailoring enzymes such as dehydrogenases, amidases and oxidases. The Pfam domain description provides functional classification for the neighbors, which will help in predicting the complete lantibiotic synthesis pathway.

## Results and benchmarking

Our benchmarking studies on completely independent datasets indicate that novPTMenzy can predict the presence of different PTM catalyzing domains with very high sensitivity and specificity (Table 1). The significance of this tool was evaluated by testing it on a set of newly sequenced hypothetical proteins. These proteins have not been used to train any of the profiles and were released within a span of 5 days, thereby forming a completely independent set. Using novPTMenzy we could identify 141 unusual PTM catalyzing domains in this set of hypothetical proteins. Of these 141, 67 were predicted to be a hydroxylase, 53 AMPylators and 22 Sulfotransferases. The complete list of these 141 proteins and their classification is provided on the ‘Benchmark’ page of novPTMenzy.

## Discussion

In summary, novPTMenzy is a unique resource for *in silico* identification and analysis of enzymes catalyzing novel/unusual PTMs. It is therefore a valuable resource for deciphering unusual PTM associated pathways by genome mining. A unique feature of novPTMenzy is the availability of HMM profiles for the identification and classification of these PTM catalyzing enzymes. novPTMenzy also contains specialized search interfaces for the prediction of inhibitory helices that regulate Fic domains and the analysis of genomic neighborhood of eliminylating enzymes. In addition to these sequence analysis tools the novPTMenzy database also provides a graphical interface for visualization of structural details of the active site pockets. Though AMPylation and Eliminylation have complete set of the features mentioned above, database and analysis tools for Deamidation, Hydroxylation and Sulfation are still in development (Table 2). Our future plan is to include more unusual PTMs in our database and to develop specific analysis tools for them. To keep our database updated, we have made provision for the inclusion of growing information about these PTMs with the participation of user community through editable pages.

**Table 2.** Features of novPTMenzy

	PTM catalyzing domain prediction	Active site Prediction	Comparative Sequence Analysis			3D Visualization (Jmol)	Other Analysis Tool	
			Closest homolog	Closest structural homolog	Phylogenetic Prediction		Search Inhibitory helix	Synteny of LanL
AMPylation	+	+	+	+	+	+	+	
Eliminylation	+	+	+	+	+	+		+
Deamidation	+		+	+	+			
Sulfation	+		+	+	+			
Hydroxylation	+		+	+	+			
